# Hepatitis B Virus Genotype-Dependent Vulnerability of Infected Cells to Immune Reaction in the Early Phase of Infection

**DOI:** 10.3389/fmicb.2019.02427

**Published:** 2019-10-18

**Authors:** Masaaki Shiina, Norie Yamada, Ryuichi Sugiyama, Asako Murayama, Hussein Hassan Aly, Masamichi Muramatsu, Takaji Wakita, Michio Imawari, Takanobu Kato

**Affiliations:** ^1^Department of Virology II, National Institute of Infectious Diseases, Tokyo, Japan; ^2^Department of Gastroenterology and Hepatology, Shin-Yurigaoka General Hospital, Kawasaki, Japan; ^3^Research Institute for Gastrointestinal and Liver Diseases, Shin-Yurigaoka General Hospital, Kawasaki, Japan

**Keywords:** hepatitis B virus, NK cell, apoptosis, innate immunity, cytotoxicity

## Abstract

Infection with hepatitis B virus (HBV) genotype (GT)-A has been reported to predispose patients to chronic infection. To explore the immune responses in infection with different HBV genotypes and clarify the genotype-dependent pathogenicity, a system mimicking the immune reaction during the early phase of HBV infection is indispensable. To this end, we established a coculture system with the replication-competent HBV molecular clone-transfected HepG2 cells and immortalized human natural killer (NK) cells, NK-92MI. Using this system, we evaluated HBV genotype dependency in NK functions and cell death of HBV positive HepG2 cells induced by NK cells or administration of tumor necrosis factor (TNF) by use of flow cytometry. After coculture with NK cells, we found that GT-A-positive HepG2 cells exhibited lower susceptibility to NK cell-induced cell death than GT-B- or GT-C-positive HepG2 cells. The NK responses of degranulation and cytokine production were not different among transfected HBV genotypes in cocultured cells. The expression levels of death receptors in HBV-transfected HepG2 cells were not different. In GT-A-positive cells, a similar low susceptibility was detected by the external administration of TNF, although relatively higher susceptibility was observed in GT-B- and GT-C-positive cells than in GT-A-positive cells. The activation of caspase signaling was revealed to be responsible for this genotype-dependent susceptibility. In conclusion, our results indicate that the HBV genotype does not influence the NK cell function itself but rather cell vulnerability through the TNF signal pathway. This observation may explain the high chronicity rate of HBV GT-A strains even in adult infections.

## Introduction

Hepatitis B virus (HBV) is a major cause of liver disease, and approximately 240 million people are infected with HBV worldwide (Schweitzer et al., 2015; Tang et al., 2018). HBV infection leads to a variety of clinical courses, e.g., acute and self-limited hepatitis, fulminant hepatitis with hepatic failure, or chronic hepatitis-promoting liver cirrhosis and hepatocellular carcinoma (Liang et al., 2015; Tang et al., 2018). Although viral hepatitis is thought to be the result of unfavorable host-immune responses against infected hepatocytes, the mechanisms to explain differences in the status or progression of hepatitis have not been clarified (Rehermann, 2013). HBV is classified into at least 9 genotypes that show different distributions among different countries, and the genotype dependency of pathogenesis and clinical courses have been indicated (Kurbanov et al., 2010; Pourkarim et al., 2014). For example, authentic genotype (GT)-B (designated GT-Bj) is related to a high rate of HBe antigen seroconversion and slow disease progression (Sugauchi et al., 2003; Ozasa et al., 2006). On the other hand, infection with GT-A results in a higher chronicity rate than infection with other genotypes (Suzuki et al., 2005; Ito et al., 2014). Therefore, the genotype dependency of the strength and/or manner of host-immune responses during HBV infection should be considered.

Natural killer (NK) cells are found more frequently in the liver than in the periphery and are thought to play an important role in viral hepatitis (Tian et al., 2013). The major function of NK cells is cytotoxicity, both through the formation of cytotoxic granules and through ligand-receptor-mediated behaviors that could be induced much earlier than the acquired immune response when helper/cytotoxic T cells mainly work (Biron, 1997). In this study, we aimed to establish a system to examine the difference in immune responses to different genotypes of HBV at the very early phase of infection. We produced an *in vitro* coculture model consisting of replication-competent HBV molecular clone-transfected HepG2 cells and an established cell line of NK cells, NK-92MI.

## Materials and Methods

### Construction of Replication Competent HBV Molecular Clones

Replication-competent HBV molecular clones were generated with sequences of patient-derived HBV. This study was carried out in accordance with the recommendations of the Ethics Committees in National Institute of Infectious Diseases (approval number is 377). The protocol was approved by the Ethics Committees. For the construction of HBV molecular clones, HBV strains from serum samples of chronic hepatitis B patients were analyzed. The total DNA in patient serum was extracted using the QIAamp Blood Mini Kit (Qiagen KK, Tokyo, Japan). The entire HBV genome was amplified by PCR with primers as previously described (Yamada et al., 2014). Amplified PCR fragments were inserted into the pGEM-T Easy vector (Promega, Madison, WI, United States), and at least 5 clones of each fragment were sequenced to determine the consensus sequence. Using the obtained fragments as templates, replication-competent HBV molecules with 1.38 × genome length were constructed (Yamada et al., 2017). Two HBV molecular clones each of GT-A, GT-B, and GT-C were prepared. The A40 and AC20 strains were generated by using HBV sequences from chronic hepatitis patients and were representative of GT-A strains. The B35 strain was generated as a representative of the GT-Bj strain isolated from a chronic hepatitis patient as reported previously (strain Bj_JPN35) (Sugiyama et al., 2006). The B18 strain was also generated by using the sequence of the GT-Bj strain isolated from a chronic hepatitis patient. As representatives of GT-C strains, previously reported strains C_JPN22 and Cpt were used and designated C22 and CCP, respectively (Sugiyama et al., 2006; Yamada et al., 2017).

### Cell Lines

We used the NK cell line NK-92MI (CRL-2408), which was obtained from the American Type Culture Collection (ATCC, Manassas, VA, United States). This cell line was established from human peripheral blood and expresses most NK cell markers except for CD16. NK-92MI cells were maintained as described on the product sheet. HepG2 cells were obtained from the European Collection of Authenticated Cell Cultures (ECACC, Salisbury, United Kingdom) and cultured in MEM supplemented with 10% fetal calf serum.

### Antibodies for Flow Cytometry

Anti-human CD3-PerCP, CD56-APC, Fas-FITC, ICAM-1-PE, MICA/B-PE, TNF-R1-PE, TNF-PE, and IFN-γ-FITC were purchased from Miltenyi Biotec (Bergisch Gladbach, Germany). Anti-human CD107a-FITC, anti-PD-L1-PE and anti-TRAIL-R1-PE were purchased from BD Biosciences (San Jose, CA, United States).

### Transfection of HBV Molecular Clones

HepG2 cells at 80–90% confluence in 100-mm dishes were transfected with 20 μg of plasmid containing the HBV molecular clone sequence using Lipofectamine 3000 Reagent (Thermo Fisher Scientific, Waltham, MA, United States) according to the manufacturer’s instructions.

### Killing Assay

NK-92MI cells were mixed with HBV-transfected HepG2 at a certain ratio in a 6-well culture plate (Corning, Corning, NY, United States) and incubated for 3 h in a 37°C, 5% CO_2_ incubator. After incubation, cells were harvested in 15 mL conical tubes, washed twice with PBS, and labeled with a blue fluorescent reactive dye (LIVE/DEAD Fixable dead cell stain kit, Invitrogen, Carlsbad, CA, United States). Labeled cells were washed once with PBS and stained with anti-CD56. To discriminate between HBV-negative and HBV-positive cells, cells labeled with LIVE/DEAD Fixable dead cell stain were permeabilized with Cytofix/Cytoperm (BD Biosciences) and further stained with rabbit anti-HBV core antigen (AB7841, Abcam, Cambridge, United Kingdom) followed by Alexa647 anti-rabbit IgG. After the final staining procedure, cells were spun down and resuspended in 500 μL of 1% paraformaldehyde-PBS. Samples were run on a FACSCalibur system (BD Biosciences) with CellQuest software. Data analysis was performed by using FlowJo ver. 8.8.7 (Tree Star, Ashland, OR, United States). These procedures were used for all of the following flow cytometric analyses.

### Degranulation Assay

Cells were harvested and resuspended at 1.25 × 10^6^ cells/ml. Using a FACS tube, the same volumes of NK-92MI and HepG2 cell suspensions were mixed gently and incubated for 4 h in a 37°C, 5% CO_2_ incubator along with anti-CD107a. After incubation, the cells were centrifuged, washed once with PBS, and stained with anti-CD3 and anti-CD56 for 15 min at room temperature in the dark.

### Quantification of NK Cell-Related Cytokines

Cells were harvested and resuspended at 1 × 10^6^ cells/ml. Using a FACS tube, the same volumes of NK-92MI and HepG2 cell suspensions were mixed gently and incubated for 4 h in a 37°C, 5% CO_2_ incubator along with 1:1,000 (v/v) GolgiPlug (BD Biosciences). After incubation, cells were stained with anti-CD3 and anti-CD56 followed by treatment with Cytofix/Cytoperm for permeabilization. Finally, cytokines were stained with anti-IFN-γ and anti-TNF for 20 min at room temperature in the dark. A cytometric bead array kit (BD Biosciences) was also used to evaluate the production of cytokines. The production of IFN-γ, TNF, granzyme B, IL-8, and MIP-1α in the coculture supernatant was measured.

### TNF Treatment and Caspase Activity Assay

The TNF-induced cell death of HBV molecular clone-transfected HepG2 cells was detected after treatment with TNF (20 ng/mL) and actinomycin D (20 ng/mL) (Saeed et al., 2011). After 24 h of treatment, dead cells were detected by staining with LIVE/DEAD Fixable dead cell stain. Caspase activation was detected by using the FAM-FLICA Assay Kit (ImmunoChemistry Technologies, Bloomington, MN, United States). After 24 h of treatment with TNF and actinomycin D, the activation of poly-caspase, caspase-8, caspase-9, and caspase-3/7 was analyzed. The caspase inhibitor was obtained from Medical and Biological Laboratories (Tokyo, Japan), and used at the concentration of 40 μM.

### Statistics

Statistical analysis was performed using one-way factorial ANOVA and multiple comparison tests by using GraphPad PRISM 7 software (GraphPad Software, La Jolla, CA, United States). *P* values <0.05 were considered to be statistically significant.

## Results

### Genotype-Dependent Susceptibility to NK Cell-Mediated Cytotoxicity

**FIGURE 1:**
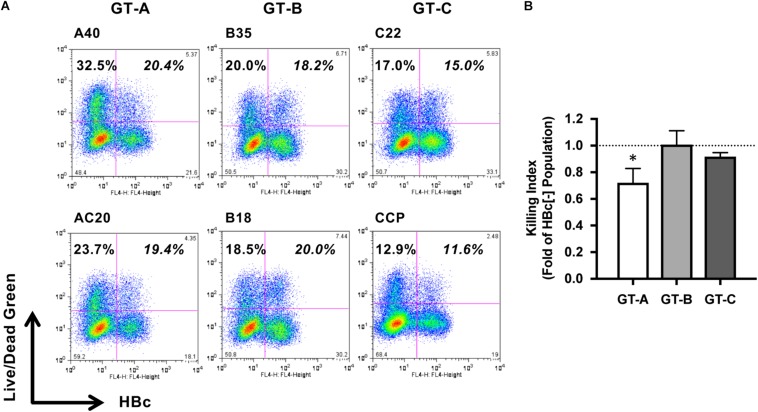
Evaluation of HBV genotype-dependent susceptibility to cytotoxicity by NK-92MI cells. **(A)** HBV genotype-dependent susceptibility to cytotoxicity by NK-92MI cells. HBV molecular clone-transfected HepG2 cells were cocultured with NK-92MI cells, and dead cells were detected by staining with LIVE/DEAD fixable dead cell staining reagent in HBV-positive and -negative populations. **(B)** The killing indexes of HBV molecular clone-transfected HepG2 cells. The killing index was calculated by dividing the percentage of dead cells in the HBV-positive population by that in the HBV-negative population. The means ± SDs of three experiments are indicated. ^∗^*P* < 0.05.

To compare the characteristics of different HBV genotypes, we generated six replication-competent HBV molecular clones, including two clones each of GT-A (A40 and AC20), GT-B (B35 and B18), and GT-C (C22 and CCP). After transfection of HepG2 cells with these HBV molecular clones, we cocultured these cells with NK-92MI cells and evaluated the HBV genotype dependency of NK cell-induced cell death. NK cell-mediated cytotoxicity was detected by staining with LIVE/DEAD Green reagent, and HBV-positive and HBV-negative populations were visualized by staining with anti-HBc and separated by flow cytometry. After the coculture of HBV-A40-transfected HepG2 cells and NK-92MI cells, we detected 20.4 and 32.5% cell death in HBV-positive and HBV-negative populations, respectively (Figure 1A). Thus, in this experiment, the susceptibility of HBV-A40-positive cells to NK cells was 0.63-fold of that exhibited by HBV-negative cells. We designated this value as the killing index. The average killing index of GT-A-transfected cells was 0.72 ± 0.11 and was significantly lower than that of GT-B- or GT-C-transfected cells (1.01 ± 0.10 and 0.92 ± 0.03, respectively) (Figure 1B). The percentages of cell death without NK cell-coculture were 5.6 ± 1.2%, 5.7 ± 1.1%, and 4.9 ± 0.9% in GT- A-, GT- B-, and GT-C-positive populations and there was no significant difference.

### Induction of NK Cell Degranulation

**FIGURE 2:**
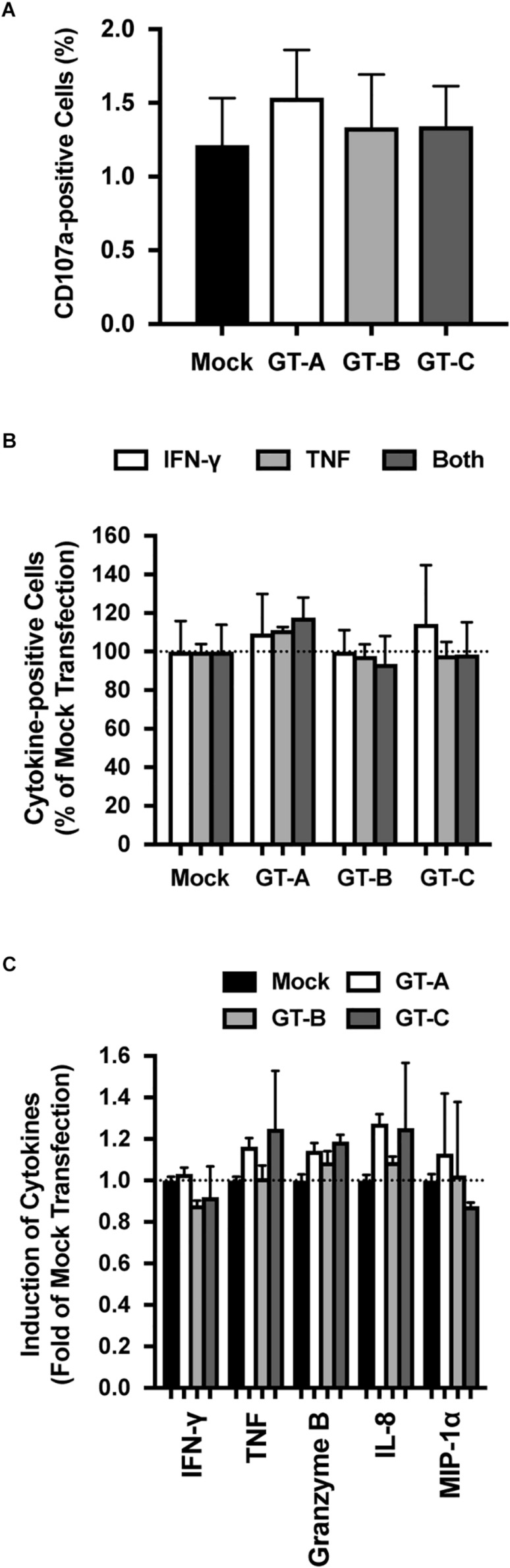
Evaluation of HBV genotype-dependent effects on NK-92MI cells. **(A)** HBV genotype-dependent effects on degranulation by NK-92MI cells. NK-92MI cells were cocultured with HBV-transfected HepG2 cells, and the degranulation induced by NK-92MI cells was detected by staining with anti-CD107a. The means ± SDs of three experiments are indicated. **(B)** HBV genotype-dependent effects on cytokine production in NK-92MI cells. NK-92MI cells were cocultured with HBV-transfected HepG2 cells, and the intracellular cytokine production of TNF, IFN-γ or both was evaluated. The means ± SDs of three experiments are indicated. **(C)** HBV genotype-dependent effects on cytokine production by NK-92MI cells. The amounts of indicated cytokines in the supernatant were quantified by cytometric bead array. The means ± SDs of three experiments are indicated.

To clarify the underlying mechanisms of genotype-dependent susceptibility to NK cell-mediated cytotoxicity, we assessed the effects of transfection with different HBV genotypes on NK cell function. We measured the CD107a-induced population of NK-92MI cells after coculture stimulation. Following incubation with mock-transfected HepG2 cells, we detected 1.07 ± 0.25% CD107a-positive cells. Following incubation with HBV-transfected HepG2 cells, the percentages of CD107a-positive cells were similar to or slightly higher than the percentage of CD107a-positive cells in the mock-transfected cells, and genotype dependency was not observed for this NK cell function (Figure 2A).

### Cytokine Production of NK Cells

We assessed cytokine production by NK-92MI cells after coculture with HBV-transfected HepG2 cells. To detect cytokine production in NK-92MI cells, these cells were permeabilized and stained with anti-interferon (IFN)-γ and anti-tumor necrosis factor (TNF) antibodies. Irrespective of the HBV genotype used to transfect the cocultured HepG2 cells, the percentages of TNF-positive NK-92MI cells, IFN-γ-positive NK-92MI cells and NK-92MI cells positive for both TNF and IFN-γ were similar (Figure 2B). The amounts of cytokine production in the culture medium was also evaluated by cytometric bead array. After coculture with HBV-transfected HepG2 cells, the levels of secreted cytokines: IFN-γ, TNF, granzyme B, interleukin (IL)-8, and macrophage inflammatory protein 1 alpha (MIP-1α) were comparable regardless of the genotype used for transfection, and genotype dependency was not observed (Figure 2C).

### Vulnerability to the Death Signal

**FIGURE 3:**
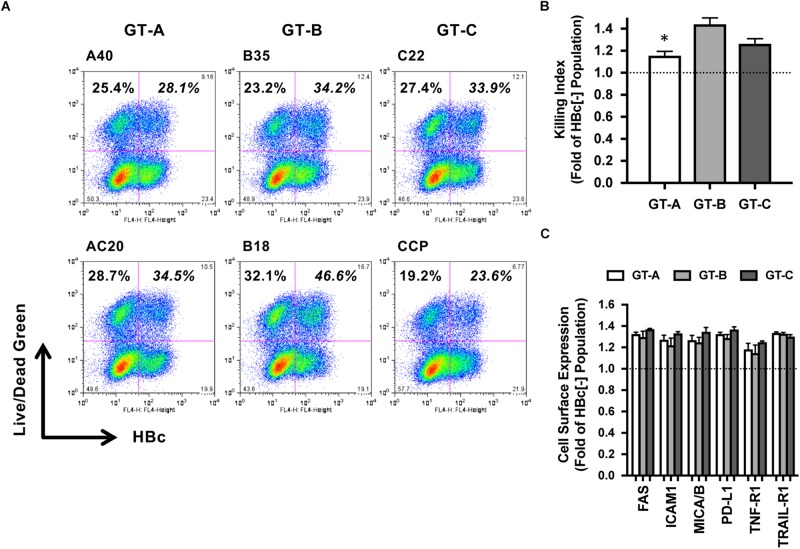
Evaluation of HBV genotype-dependent cytotoxicity by treatment with TNF. **(A)** HBV genotype-dependent effects on cytotoxicity by treatment with TNF. HBV-transfected HepG2 cells were treated with TNF, and dead cells were detected by staining with LIVE/DEAD fixable dead cell staining reagent. HBV-positive and -negative populations were separated after staining with anti-HBc. **(B)** Killing indexes of HBV-transfected HepG2 cells. The killing index was calculated by dividing the percentage of dead cells in the HBV-positive population by that in the HBV-negative population. The means ± SDs of three experiments are indicated. ^∗^*P* < 0.05. **(C)** HBV genotype-dependent effects on the cell surface expression of cell death-associated receptors. Effects on the expression levels of the indicated receptors are indicated as ratios calculated by dividing the mean fluorescence intensity in the HBV-positive population by that in the HBV-negative population.

As a next step, we evaluated the genotype dependency of susceptibility to the cell death signal of HBV-transfected HepG2 cells. TNF is a well-known cytotoxic effector produced by NK cells. Therefore, cell death induced by TNF administration was examined. Among the two GT-A-positive populations, TNF-induced cell deaths were detected in 28.1 and 34.5% of cells, respectively, and were slightly higher than those among the two HBV-negative cell populations (25.4 and 28.7%, respectively) (Figure 3A). The average killing index of GT-A-transfected cells was 1.16 ± 0.04, which was significantly lower than that of GT-B- or GT-C-transfected cells (1.44 ± 0.06 and 1.27 ± 0.05, respectively) (Figure 3B). We evaluated the cell surface expression of cell death-associated receptors including FAS, intercellular adhesion molecule-1 (ICAM-1), major histocompatibility complex class I-related chain A and B (MICA/B), programed cell death-ligand 1 (PD-L1), TNF-R1, and TNF-related apoptosis-inducing ligand (TRAIL-R1) by flow cytometry. The expression levels of these molecules were upregulated in HBV-positive populations compared to those in HBV-negative populations, but genotype-dependent differences were not found (Figure 3C).

### Genotype-Dependent Caspase Activation by TNF

**FIGURE 4:**
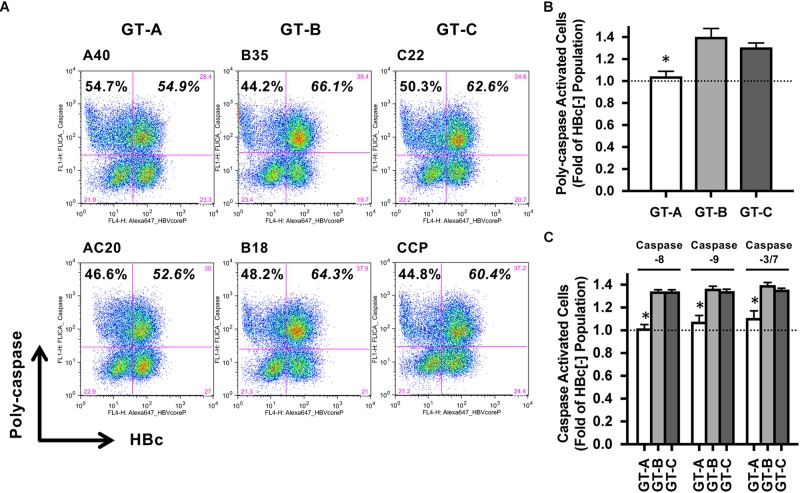
Evaluation of HBV genotype-dependent caspase activation by treatment with TNF. **(A)** HBV genotype-dependent effects on poly-caspase activation by treatment with TNF. HBV-transfected HepG2 cells were treated with TNF, and poly-caspase-activated cells were detected by staining with FAM-FLICA reagent in HBV-positive and -negative populations. **(B)** Ratios of poly-caspase-activated cells in HBV-transfected HepG2 cells. The ratio was calculated by dividing the percentage of poly-caspase-activated cells in the HBV-positive population by that in the HBV-negative population. The means ± SDs of three experiments are indicated. ^∗^*P* < 0.05. **(C)** Ratios of caspase-activated cells in HBV molecular clone-transfected HepG2 cells. The ratios of caspase- 8-, caspase-9- or caspase-3/7-activated cells were calculated by dividing the percentage of caspase-activated cells in the HBV-positive population by that in the HBV-negative population. The means ± SDs of three experiments are indicated. ^∗^*P* < 0.05.

To assess the HBV genotype dependency of susceptibility to the TNF-induced death signal, we analyzed the activation of caspase signaling on a single-cell level. After treatment with TNF, the proportion of poly-caspase-activated cells in the GT-A-positive populations was similar to that in the HBV-negative populations. On the other hand, the proportion of poly-caspase-activated cells in the GT-B- or GT-C-positive populations was substantially higher than those in the HBV-negative populations (Figure 4A). The ratio of the proportion of poly-caspase-activated cells in the GT-A-positive population to the GT-A-negative population was 1.04 ± 0.05. This ratio was significantly lower than the cases of GT-B or GT-C-transfection (1.40 ± 0.08 and 1.31 ± 0.04, respectively) (Figure 4B). This genotype-dependent activation of poly-caspase was also confirmed in the coculture system with the replication-competent HBV molecular clone-transfected HepG2 cells and NK cells (Supplementary Figure S1). To determine the first caspase affected in the caspase cascade, we assessed 3 caspases: caspase-8, caspase-9, and caspase-3/7. The proportion of caspase-8-activated cells in GT-A-transfected cells was significantly lower than that in cells transfected with other genotypes, and similar observations were demonstrated in assays determining the proportion of caspase-9- and caspase-3/7-activated cells (Figure 4C). Finally, to confirm the involvement of caspase activation on apoptosis induction, we assessed the impact of the caspase inhibitor on the ratios of TNF-induced cell death. By treatment with the caspase inhibitor, the percentages of caspase-activated cells by administration of TNF were substantially reduced than without the caspase inhibitor. However, even in such a situation, the genotype-dependency on the ratios of caspase-activated cells was maintained (Supplementary Figure S2).

## Discussion

Once chronic infection with HBV is established, HBV cannot be eradicated by currently available treatment with nucleos(t)ide analogs and/or exogenous interferons (Liang et al., 2015; Tang et al., 2018). To explore novel anti-HBV treatments that can eradicate HBV infection, it is important to clarify the underlying mechanisms for the establishment of chronicity. *Ex vivo* studies have shown that HBV-specific immune responses are exhausted in chronically infected patients compared to those in patients with acute and self-limited infection (Oliviero et al., 2009; Peppa et al., 2010; Martinet et al., 2012). It is still difficult to predict clinical outcomes of HBV infection either by analyzing the strength of HBV-specific immune responses or by inflammation levels. Assessment of the early immune response to HBV infection is known to be important; however, it is difficult to obtain materials from patients in this phase of hepatitis who show little or no symptoms. Experimental infection in the chimpanzee model may be able to overcome this problem (Wieland et al., 2004), but the strength of the induced anti-HBV immune responses in the early phase of hepatitis and their effect on the clinical outcomes of HBV infection have not yet been clarified in this model. Moreover, experiments using the chimpanzee model are no longer permitted. Here, to explore early immune responses before the establishment of acquired immunity and specific antibodies, we established an *in vitro* coculture model consisting of HBV molecular clone-transfected HepG2 cells and an established NK cell line. This system is expected to mimic the first NK cell response against HBV-infected hepatocytes in the liver and to enable investigation of genotype-dependent effects on NK cell-mediated cytotoxicity.

We examined the killing activity of NK cells against HepG2 cells transfected with HBV clones of different genotypes. In this study, we used a fine flow cytometric technique to detect dead cells in HBV-positive and HBV-negative populations by costaining and found that the vulnerability of HBV GT-A-positive cells to NK cell-mediated cytotoxicity was lower than that of GT-B- or GT-C-positive cells. We confirmed that the percentages of cell death of HBV-positive cells without NK-cell coculture were the same levels among these genotypes in this system, although the frequent induction of apoptosis of GT-C-positive cells has been reported in the immunodeficient NOG mice (Yokoyama et al., 2015). To investigate whether NK cells behave differently depending on HBV genotype in target cells, we examined typical NK cell functions: CD107a degranulation, intracellular IFN-γ/TNF synthesis, and the production of inflammatory cytokines. However, no apparent differences were detected in these NK cell functions irrespective of the HBV genotype used to transfect the target cells. Therefore, we reasoned that the observed genotype-dependent susceptibility to NK cell-mediated cytotoxicity does not depend on NK cell function. Thus, we evaluated the effects of different HBV genotypes used to transfect target cells. To compare susceptibilities to death signal activation by NK cells, the HBV-transfected cells were treated with TNF, a well-known cytotoxic effector produced by NK cells, and evaluated for induced cytotoxicity. The percentage of dead cells were remarkably higher in GT-B- and GT-C-transfected cells than in GT-A-transfected cells. The cell surface expression of death receptors including TNF-R1 was evaluated, but no difference was detected among cells transfected with the HBV genotypes. Taken together, we concluded that the genotype-dependent difference in cytotoxicity observed in the coculture model with NK cells can be ascribed to susceptibility to TNF-induced cell death, but this difference was not attributable to the cell surface expression of death receptors. To explore the underlying mechanisms of genotype-dependent susceptibility to TNF, we examined caspase activation in HBV-transfected HepG2 cells treated with TNF. The percentage of poly-caspase-activated cells was higher in GT-B- and GT-C-transfected cells than in GT-A-transfected cells. This lower susceptibility was detected for the activation of caspase-8, caspase-9 and caspase-3/7. Taken together, HBV GT-A infection inhibited the early step of apoptosis induction by NK cells without any effect on their function.

There are some limitations to our study. First, we used only one combination of effector cells and target cells: NK-92MI cells and HBV-transfected HepG2 cells. NK-92MI cells are from a self-replicating tumorous cell line that does not express CD16, which is an important Fc receptor for antibody-dependent cellular cytotoxicity (ADCC). Concerning NK cell function, we cannot evaluate ADCC in this system. However, this could be ignored during this study because we focused on NK cell-mediated cytotoxicity against non-self-antigens at the early phase of infection. To detect the effects of HBV infection on NK cell function, a combination of human peripheral blood mononuclear cells (PBMC) and primary hepatocytes is also pertinent. However, the frequency and activation strength of NK cells varied greatly between individuals, and primary hepatocytes were fragile and not stable enough for transfection and/or killing assays. Thus, this strategy is not suitable for studies that require reproducibility. Second, we only used two clones per HBV genotype, which may not be sufficient to explain all of the evidence related to the genotypic differences. Third, we could not specify the molecule(s) and effector site of the inhibitory signal in our system. Since the activity of caspase-8, which is part of the early steps of the intracellular cascade producing an apoptotic signal, is affected, changes of inhibitory signaling molecules, such as TNF receptor-associated factors (TRAFs), inhibitors of apoptosis protein (IAPs), or FLICE-inhibitory proteins (FLIP), might play a role (Deveraux et al., 1998; Wang et al., 1998; Matsuda et al., 2014; Ebert et al., 2015). This point should be addressed using a refined apoptosis detection model in the future.

## Conclusion

In conclusion, to evaluate immune responses in the early phase of HBV infection, we established an *in vitro* model system by using an NK cell line and HepG2 cells transfected with different HBV strains. Using this system, we found that the HBV GT-A-positive HepG2 cells exhibited lower susceptibility to NK cell- or TNF-induced apoptosis than GT-B- and GT-C-positive cells. This observation may be associated with the high chronicity rate seen for infection with HBV GT-A strains even in adult infections.

## Data Availability Statement

All datasets generated for this study are included in the manuscript/Supplementary Files.

## Author Contributions

MS and TK designed the concepts of this study, discussed and interpreted the results, and wrote the manuscript. MS, NY, RS, AM, HA, and TK carried out the experiment. MM, TW, and MI supervised the experiment and project.

## Conflict of Interest

The authors declare that the research was conducted in the absence of any commercial or financial relationships that could be construed as a potential conflict of interest.

## Abbreviations

ADCC: antibody-dependent cellular cytotoxicity
FLIP: FLICE-inhibitory proteins
GT: genotype
HBV: hepatitis B virus
IAPs: inhibitors of apoptosis protein
ICAM-1: intercellular adhesion molecule-1
IFN: interferon
IL: interleukin
MHC: major histocompatibility complex
MICA/B: major histocompatibility complex class I-related chain A and B
MIP-1 α: macrophage inflammatory protein 1 alpha
NK: natural killer
PBMC: peripheral blood mononuclear cells
PD-L1: programed cell death-ligand 1
PMA: phorbol 12-myristate 13-acetate
TNF: tumor necrosis factor
TRAFs: TNF receptor-associated factors
TRAIL: TNF-related apoptosis-inducing ligand.
