# Supplementary material for: Hepatitis B Virus Genotype-Dependent Vulnerability of Infected Cells to Immune Reaction in the Early Phase of Infection
Source: Front Microbiol. 2019 Oct 18;10:2427. doi: 10.3389/fmicb.2019.02427 (PMC6813626; doi:10.3389/fmicb.2019.02427)
Supplement: Supplementary file 1 [file Data_Sheet_1.PDF]

SUPPLEMENTAL MATERIALS

Hepatitis B Virus Genotype-dependent Vulnerability of Infected Cells to Immune Reaction in the Early Phase of Infection

Masaaki Shiina, Norie Yamada, Ryuichi Sugiyama, Asako Murayama,

Hussein Hassan Aly, Masamichi Muramatsu, Takaji Wakita,

Michio Imawari, Takanobu Kato.

SUPPLEMENTARY FIGURE LEGENDS

Supplementary Figure 1. Evaluation of HBV genotype-dependent poly-caspase activation by NK-92MI cells.

(A) HBV genotype-dependent poly-caspase activation by NK-92MI cells. HBV molecular clones-transfected HepG2 cells were cocultured with NK-92MI cells, and poly-caspase-activated cells were detected by staining with FAM-FLICA reagent in HBV-positive and -negative populations. (B) Ratios of poly-caspase-activated cells in HBV-transfected HepG2 cells. The ratio was calculated by dividing the percentage of poly-caspase-activated cells in the HBV-positive population by that in the HBV-negative population. The means ± SDs of three experiments are indicated. $^*P < 0.05$.

Supplementary Figure 2 Effects of caspase inhibitor on HBV genotype-dependent caspase activation.

(A) HBV genotype-dependent caspase 3/7 activation by treatment

with TNF. HBV molecular clones-transfected HepG2 cells were treated with TNF with or without the caspase inhibitor, and caspase 3/7-activated cells were detected by staining with FAM-FLICA reagent in HBV-positive and -negative populations. (B) Ratios of caspase 3/7-activated cells in HBV-transfected HepG2 cells. The ratio was calculated by dividing the percentage of caspase 3/7-activated cells in the HBV-positive population by that in the HBV-negative population. The means ± SDs of three experiments are indicated. *$P < 0.05$.
