# Supplementary figures and images for: Hepatitis B Virus Genotype-Dependent Vulnerability of Infected Cells to Immune Reaction in the Early Phase of Infection
Source: Front Microbiol. 2019 Oct 18;10:2427. doi: 10.3389/fmicb.2019.02427 (PMC6813626; doi:10.3389/fmicb.2019.02427)

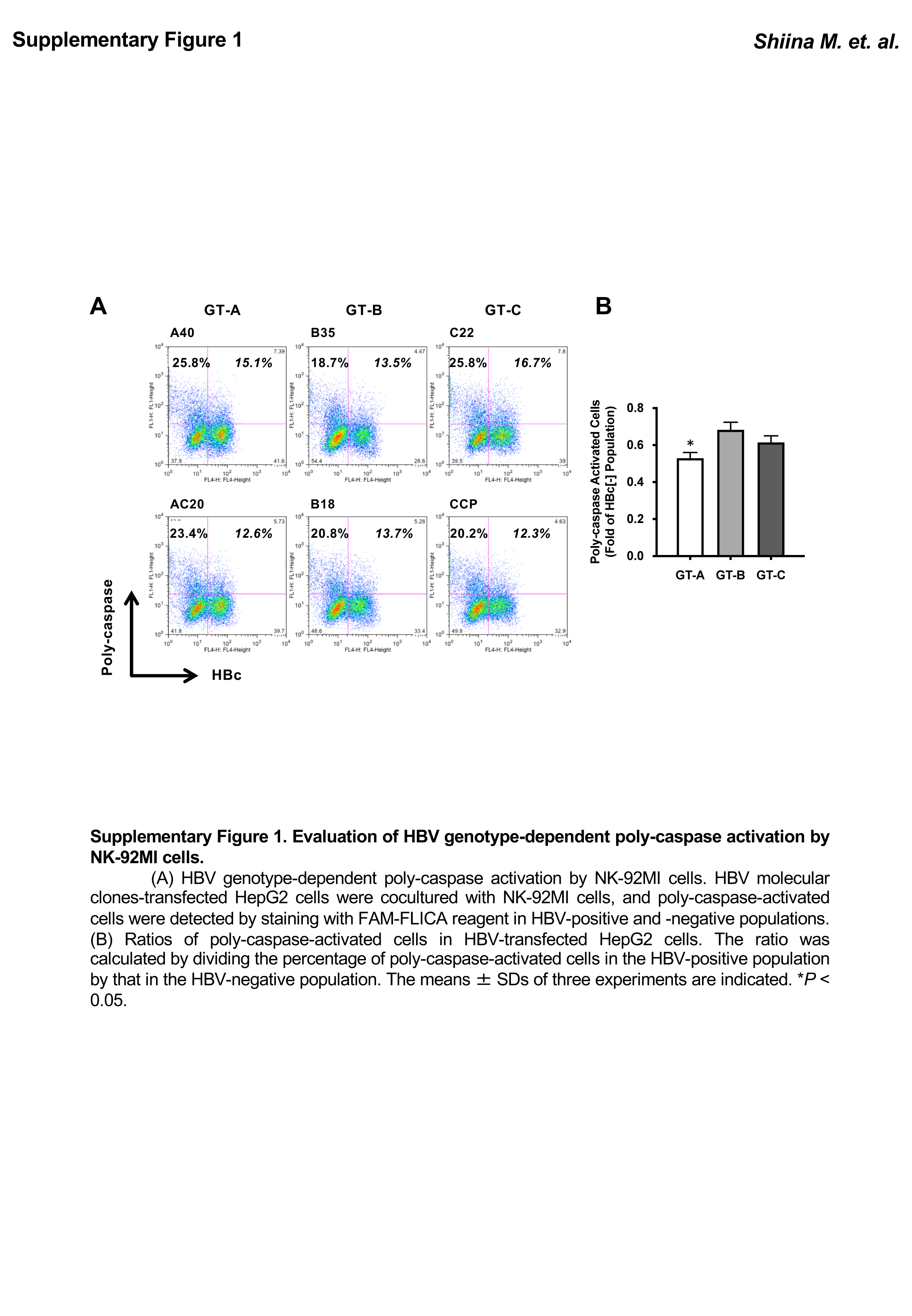

Supplement: Supplementary file 2 [file Image_1.TIF]

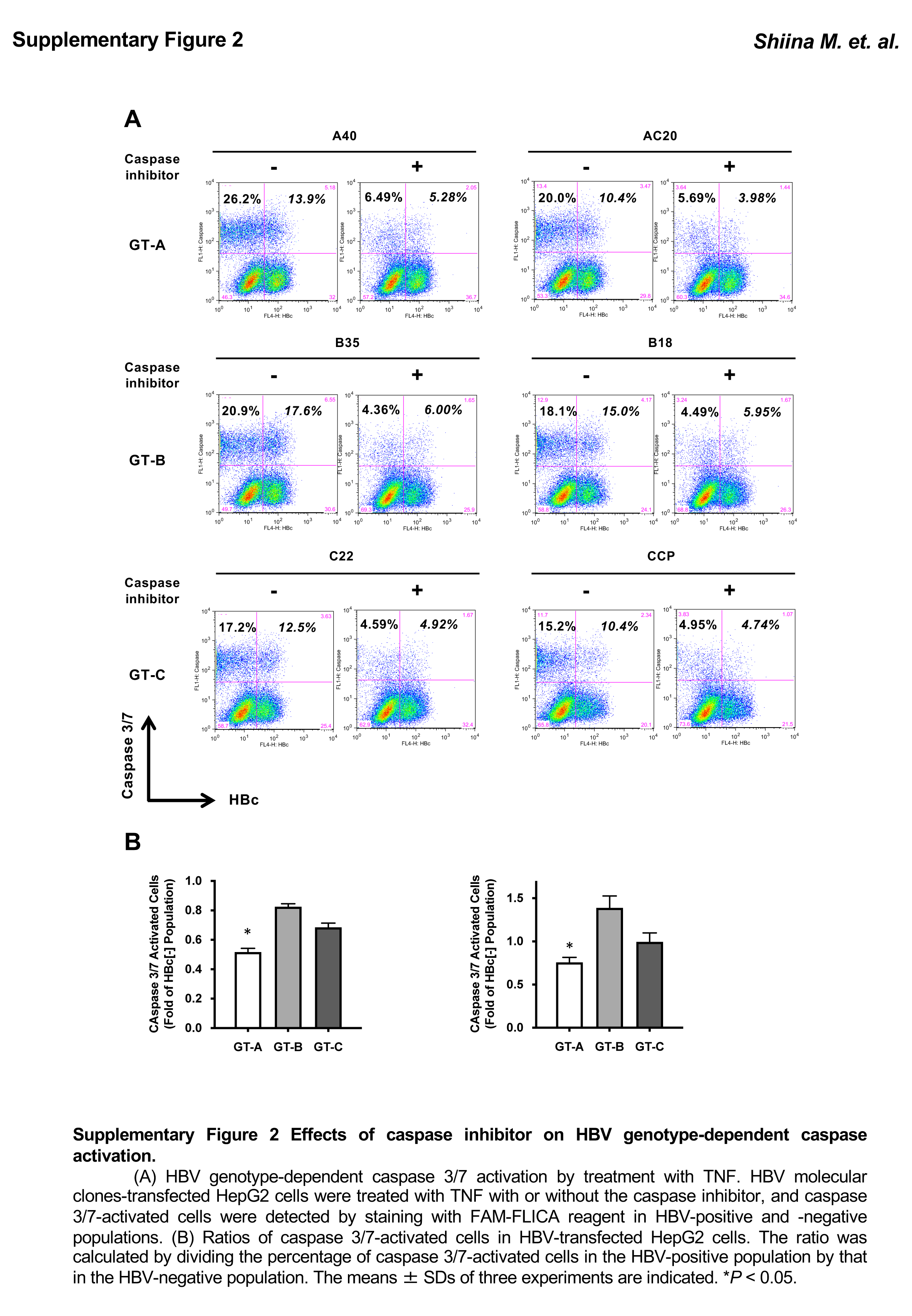

Supplement: Supplementary file 3 [file Image_2.TIF]
